# Trauma Team Activation: Which Surgical Capability Is Immediately Required in Polytrauma? A Retrospective, Monocentric Analysis of Emergency Procedures Performed on 751 Severely Injured Patients

**DOI:** 10.3390/jcm10194335

**Published:** 2021-09-23

**Authors:** Daniel Schmitt, Sascha Halvachizadeh, Robin Steinemann, Kai Oliver Jensen, Till Berk, Valentin Neuhaus, Ladislav Mica, Roman Pfeifer, Hans Christoph Pape, Kai Sprengel

**Affiliations:** 1 Department of Trauma, University Hospital Zurich (USZ), Raemistrasse 100, 8091 Zurich, Switzerland; sascha.halvachizadeh@usz.ch (S.H.); robin.steinemann@uzh.ch (R.S.); kaioliver.jensen@usz.ch (K.O.J.); till.berk@usz.ch (T.B.); valentin.neuhaus@usz.ch (V.N.); ladislav.mica@usz.ch (L.M.); roman.pfeifer@usz.ch (R.P.); hans-christoph.pape@usz.ch (H.C.P.); kai.sprengel@uzh.ch (K.S.); 2 Faculty of Medicine, University of Zurich (UZH), Raemistrasse 71, 8006 Zurich, Switzerland; 3 Hirslanden Clinic St. Anna, St. Anna-Strasse 32, 6006 Lucerne, Switzerland

**Keywords:** polytrauma, emergency surgery, trauma team competence, trauma system, life-saving intervention

## Abstract

There has been an ongoing discussion as to which interventions should be carried out by an “organ specialist” (for example, a thoracic or visceral surgeon) or by a trauma surgeon with appropriate general surgical training in polytrauma patients. However, there are only limited data about which exact emergency interventions are immediately carried out. This retrospective data analysis of one Level 1 trauma center includes adult polytrauma patients, as defined according to the Berlin definition. The primary outcome was the four most common emergency surgical interventions (ESI) performed during primary resuscitation. Out of 1116 patients, 751 (67.3%) patients (male gender, 530, 74.3%) met the inclusion criteria. The median age was 39 years (IQR: 25, 58) and the median injury severity score (ISS) was 38 (IQR: 29, 45). In total, 711 (94.7%) patients had at least one ESI. The four most common ESI were the insertion of a chest tube (48%), emergency laparotomy (26.3%), external fixation (23.5%), and the insertion of an intracranial pressure probe (ICP) (19.3%). The initial emergency treatment of polytrauma patients include a limited spectrum of potential life-saving interventions across distinct body regions. Polytrauma care would benefit from the 24/7 availability of a trauma team able to perform basic potentially life-saving surgical interventions, including chest tube insertion, emergency laparotomy, placing external fixators, and ICP insertion.

## 1. Introduction

Trauma is among the leading causes of morbidity and mortality in the working population [1]. Prehospital, a differentiated triage system of severely injured patients’ increased survival rates [2,3] have led to the development of trauma centers, trauma networks, and national trauma registries [4,5,6]. Furthermore, the deployment of trauma teams has constantly improved survival [7,8]. Local institutional trauma guidelines define the members of the trauma team and the algorithms for trauma team activation [9,10]. Selected institutions have the luxury of activating an interdisciplinary trauma team, including an anesthesiologist, radiologist, neurosurgeon, and a trauma surgeon with surgical competences of the whole body [7].

There has been an ongoing discussion, especially in German speaking countries, as to which level of thoracoabdominal interventions a general surgeon is capable of providing as primary care to polytrauma patients, especially due to changes in the training of medical specialists [11,12]. In particular, whether thoracoabdominal interventions should be performed by an “organ specialist” (for example, a thoracic or visceral surgeon) who is present in the resuscitation area, or by a trauma surgeon with the appropriate general surgical training and the skills required for damage control surgery [13]. The principle of interdisciplinary collaboration under the direction of a general trauma surgeon is currently the basis of major trauma centers [14].

Unfortunately, there is only limited information about the life-saving surgeries that are immediately carried out.

To improve trauma systems and training adequately, an overview of the most common emergency interventions for severely injured patients is required. Therefore, the aim of this study was to describe the most commonly performed emergency surgical interventions (ESI) on polytrauma patients and their impact on morbidity and mortality.

## 2. Materials and Methods

This retrospective cohort study strictly follows the Strengthening the Reporting of Observational Studies in Epidemiology (STROBE) Statement [15].

This study was conducted at one academic Level 1 trauma center, utilizing a retrospective database of polytrauma patients. The database included demographics, injury severity and distribution, vital parameters, laboratory values that were routinely assessed during medical treatment, in-hospital mortality, and complications, with a follow-up of 30 days. All patients received a whole-body computed tomography (WBCT) upon arrival in the trauma bay.

This study includes data of polytrauma patients over a period of 16 years who fulfilled the criteria of the Berlin definition of polytrauma. Furthermore, patients with data regarding type of ESI, injury distribution, and complications were included. Secondarily transferred patients, patients with end-of life treatment, or patients with a signed “do not resuscitate” (DNR) form were excluded. The study population was stratified into patients requiring emergency surgical interventions (Group ESI) and patients without emergency surgical intervention (Group non-ESI).

The primary outcome of this study was to describe the four most common ESI and their impact on the course of the polytrauma patient. ESI included all surgical interventions that were performed within 24 h of admission. The four most common ESI were summarized, including each of the most relevant specific procedures. Injury severity was measured utilizing the ISS [16], while injury distribution and local injury severity were stratified according the Abbreviated Injury Scale (AIS) [17]. This study only analyzed injuries with an AIS of 3 or higher. The neurological status was assessed using the Glasgow Coma Scale (GCS) [18].

The course of the polytrauma patients was assessed by in-hospital mortality and 30-day major complications, including pneumonia, sepsis, bacteremia, and infections requiring medical or surgical treatment. All variables were collected during routine medical treatment of the polytrauma patients. The vital parameters and laboratory results were measured on arrival, while AIS and ISS were calculated based on the information given on the patients discharge papers. An a-priori sample size calculation was not warranted since this study analyzed maximum available datasets. The vital parameters and laboratory results were chosen in reference to the Berlin definition of polytrauma.

Data were tested for normal distribution using the Shapiro–Wilk normality test. Continuous variables were summarized as median and interquartile range (IQR, 25th–75th percentile). Categorical variables were displayed with count and percentage. Group comparisons with two partners of continuous variables was performed using the Student’s *t*-test (normal distribution) or the Mann–Whitney U-test (skewed distribution). Comparisons of categorical variables were performed using the Pearson Chi-squared test. Statistical significance was set at a *p*-value of <0.05. All calculations were performed using R Core Team (2018) (R:A language and environment for statistical computing, R Foundation for Statistical Computing, Vienna, Austria, URL: https://www.R-project.org/ (accessed on 16 August 2021)).

## 3. Results

### 3.1. Study Population

**Figure 1:**
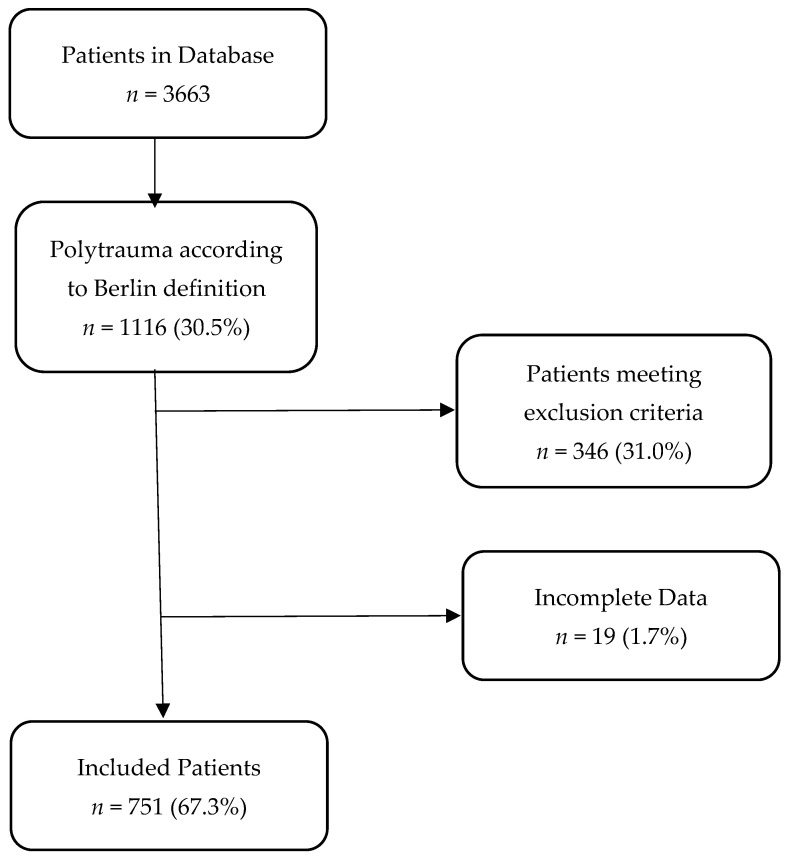
Flowchart of patient selection.

The utilized database contains the records of 3663 patients, with 1116 (30.5%) patients meeting the Berlin definition of polytrauma. After removing the patients that were secondarily transferred, received end-of life treatment, or presented with missing data, 751 (67.3%) patients were included in our study (Figure 1).

The median age of the study population was 39 years (25, 48), with 558 (74.3%) patients being male. The median ISS was 38 (29, 45 (25th, 75th percentile)) and patients had a median GCS of 3 (3, 12) points. The median entry lactate level of all included patients was 3 mmol/l (2, 5), and the median entry arterial pressure (MAP) of all included patients was 87 mmHg (70, 100). Group ESI included 711 patients (94.5%) and Group non-ESI had 40 (5.5%) (Table 1).

### 3.2. Injury Mechanism, Severity, and Distribution

The most common injury mechanism was a motor vehicle accident (*n* = 447, 59.5%). In total, 2238 injuries with an AIS of 3 or higher were documented. The most common injury with an AIS of 3 or higher was that at the thorax (*n* = 591, 26.4%), followed by the head (*n* = 535, 23.9%), the extremities (*n* = 351, 15.7%) and the abdomen (*n* = 312, 13.9%).

Group ESI included patients with significantly higher AIS head (*p* < 0.001), AIS abdomen (*p* = 0.007), and AIS extremity (*p* = 0.007). The AIS for the face, thorax, spine, pelvis, and integument were similarly distributed among these groups (Table 2).

### 3.3. Most Common ESI

In total, 69 different surgical interventions were performed in our study population, and 832 surgical interventions were documented in the database. Out of these, the most common ESI were emergency thoracotomy (*n* = 341, 41%), followed by damage-control laparotomy (*n* = 187, 22.5%), the external fixation of an extremity (*n* = 167, 20.1%), and insertion of an ICP monitor (*n* = 137, 16.5%) (Table 3). More elaborate surgical procedures such as lung wedge resection (*n* = 7) or nephrectomy (*n* = 6) were not taken into account, as they were very rare in the observed timespan.

### 3.4. Complications

In total, complications such as infection, pneumonia, sepsis, or bacteremia were documented 783 times. The mortality of the included study population was 34.4%. The most common cause of death was traumatic brain injury (*n* = 127, 49.0%), followed by hemorrhagic shock (*n* = 82, 31.7%), multiple organ failure and systemic inflammatory response syndrome (SIRS) (*n* = 37, 14.3%), and others (*n* = 13, 5.0%). The most common complications were infection (*n* = 309, 39.5%), followed by pneumonia (*n* = 207, 26.4%), sepsis (*n* = 179, 22.9%), and bacteremia (*n* = 88, 11.2%). The rate of complications in Group ESI versus Group non-ESI was comparable (44.1% vs. 45.7% p = n.s.). Furthermore, the distribution of the rate of each assessed complication was comparable among those groups (Table 4).

## 4. Discussion

Polytrauma management substantially benefits from interdisciplinary teamwork, with an experienced leader heading the group. However, the specific training and medical education required of the trauma team members is still controversially discussed. The aim of this study was to summarize the most common surgical emergency interventions for polytrauma patients and to further analyze the impact of ESI on morbidity and mortality. This study revealed the following points:

1. Most polytrauma patients required an emergency surgical intervention within 24 h of admission;
2. Chest tube insertion, damage-control laparotomy, placing an external fixator on the extremities, and insertion of an intracranial pressure probe accounted for the most common potentially life-saving emergency surgical interventions;
3. Morbidity and mortality were not affected by emergency surgical interventions.

The distribution of injury severity is comparable to other hospitals in Western Europe [19,20].

The study population of this study represent “borderline” or “in extremis” polytrauma cases [21,22,23]. The presented mortality is comparable to current literature, where a mortality of 15–40% is described for patients that count as intermediate or high-risk according to the PolyTrauma Grading Score [24]. A multicenter study of The UK National Surgical Collective from 2017 found that 21.7% of patients who had general surgery developed sepsis, which is in the range of our results [25]. In current literature, the most commonly performed damage-control surgery on the trunk is laparotomy for abdominal packing at 56.5% [26]. Approximately 30% of penetrating and up to 15% of blunt-chest trauma require surgical treatment via thoracotomy or thoracoscopy, excluding the insertion of chest tubes alone [27]. Regarding chest tube insertion, there are rates of up to 93% for chest tube insertion in blunt thoracic trauma described in [28].

Following this definition, some sort of ESI are warranted. Furthermore, the current study population showed pathophysiologic relevant changes that are associated with the requirement of life-saving interventions [29]. While the role and strategies of fracture fixation in polytrauma have been described in numerous studies [30], only a few studies have investigated strategies for surgical interventions in the thorax and abdomen that exceeded the damage-control approach [31]. A growing body of literature has investigated damage-control principles, both in abdominal trauma [32,33] and thoracic trauma [28,34], and their effect on the outcome of polytrauma patients. It appears evident that the adequate treatment of thoracic and abdominal injuries is equally important as the treatment of fractures. Current medical advancements encourage minimal invasive procedures to control hemorrhage [35]. An increasing number of traumatic hepatic and splenic injuries are treated non-operatively [36,37] or with the support of interventional radiology (e.g., coiling) [38,39]. With evolving minimally invasive techniques or the non-operative treatment of solid organ lesions, there might be a higher threshold for the indication to perform damage-control laparotomy [40,41].

### Limitations

In the utilized database, there was only limited information about non-operative procedures. Nevertheless, the use of interventional radiologic procedures is an important topic in relation to polytrauma patients and is a part of future research in our trauma center.

There is a significant difference of age between Group ESI and non-ESI that we cannot explain with our study. This finding might be due to different trauma mechanisms. However, the ISS of both groups is similar. One might explain it with the calculation of the ISS, since it is calculated according to different regions of the body; it is not possible to distinguish between multiple injuries of one body region and it does not indicate the need for emergency surgery.

Patients who received an external fixation of long bone fractures might have required this intervention due to severe soft tissue damage, but are included in Group ESI. One might argue that the placement of an external fixator is not always a life-saving emergency surgical intervention. However, we feel that this intervention might improve the outcome of polytrauma patients who are in extremis, or in stable patients with deranged soft tissue [42].

In our Level 1 trauma center, an emergency surgical intervention is usually executed by a general surgeon; only an intracranial pressure probe is performed by a neurosurgeon. If morbidity and mortality change, whether a general surgeon or an organ specialist performs the emergency surgical intervention cannot be answered with our database.

This was a single-center study conducted at a Level 1 trauma center with more seriously injured patients compared to smaller hospitals. Moreover, there are different systems and different approaches for treating polytraumatized patients in the resuscitation area. We still think that the results are interesting for other major trauma systems to use in the training of medical personnel in the resuscitation area, with focus on the ESI.

## 5. Conclusions

Polytrauma patients often require surgical emergency intervention within the first 24 h after admission. The most commonly performed emergency procedures include thoracotomy, emergency laparotomy, external fixation of fractures of an extremity, and the insertion of an intracranial pressure probe. Polytrauma management would benefit from round-the-clock expertise in these most potentially life-saving interventions, with a limited variety provided by either an on-call “organ specialists” or a capable trauma team member with knowledge in general surgery.

## Figures and Tables

**Table 1:** Demographics and injury description of the study population.

	ESI	Non-ESI	*p*-Value
*n*	711	40	
Age (years)	38.0 (25, 56)	62 (43, 75)	<0.001
Male, *n* (%)	530 (74.5)	28 (70.0)	n.s.
ISS (points)	38 (29, 45)	34 (27, 38)	0.043
GCS (points)	7 (3, 13)	11 (6, 14)	<0.001
Lactate admission (mmol/L)	3 (2, 5)	3 (1, 4)	n.s.
MAP admission (mmHg)	85 (70, 100)	95 (75, 107)	n.s.
Heartrate admission (1/min)	100 (84, 115)	85 (74, 104)	0.011
Hematocrit admission (%)	30 (22, 36)	36 (32, 39)	<0.001
Hemoglobin admission (g/L)	10 (8, 12)	12 (10, 14)	0.015
Base excess admission (mmol/L)	−6/−9, −3)	−5 (−7, −2)	0.104
Body temperature (°C)	35 (34, 36)	35 (35, 37)	0.49

*n* = number; ESI = Emergency Surgical Intervention; ISS = Injury Severity Score; GCS = Glasgow Coma Scale; MAP = Mean Arterial Pressure; n.s. = not significant.

**Table 2:** Comparison of injury severity and injury distribution according to AIS.

	ESI	Non-ESI	
*n*	711	40	
AIS Head (points), *n* (%)			<0.001
3	122 (17.2)	12 (30.0)	
4	149 (21.0)	7 (17.5)	
5	228 (32.1)	7 (17.5)	
6	6 (0.8)	4 (10.0)	
AIS Face (points), *n* (%)			0.075
3	72 (10.2)	10 (25.0)	
4	27 (3.8)	1 (2.5)	
5	2 (0.3)	0 (0.0)	
AIS Thorax (points), *n* (%)			0.723
3	368 (51.8)	23 (57.5)	
4	137 (19.3)	5 (12.5)	
5	54 (7.6)	2 (5.0)	
6	2 (0.3)	0 (0.0)	
AIS Abdomen (points), *n* (%)			0.007
3	72 (0.2)	8 (20.0)	
4	139 (19.7)	2 (5.0)	
5	90 (12.7)	1 (2.5)	
AIS Spine (points), *n* (%)			0.464
3	95 (13.5)	8 (20.0)	
4	11 (1.6)	0 (0.0)	
5	23 (3.3)	0 (0.0)	
6	1 (0.1)	0 (0.0)	
AIS Extremity (points), *n* (%)			0.007
3	249 (35.4)	6 (15.0)	
4	66 (9.4)	1 (2.5)	
5	31 (4.4)	0 (0.0)	
AIS Pelvis (points), *n* (%)			0.318
3	117 (6.7)	7 (17.5)	
4	28 (4.0)	0 (0.0)	
5	15 (2.1)	0 (0.0)	
AIS Integument (points), *n* (%)			0.055
3	20 (2.9)	1 (2.5)	
4	6 (0.9)	0 (0.0)	
5	2 (0.3)	1 (2.5)	

AIS = Abbreviated Injury Scale; ESI = Emergency Surgical Intervention.

**Table 3:** The most common surgical interventions within 24 h.

Emergency Thoracotomy, *n* (%)		341 (41.0)
	Chest tube	191 (56.0)
	Open CPR	35 (10.3)
	Thoracic packing	24 (7.0)
Emergency Laparotomy, *n* (%)		187 (22.5)
	Abdominal packing	98 (52.4)
	Splenectomy	63 (33.7)
	Pelvic packing	34 (18.2)
External Fixation, *n* (%)		167 (20.1)
	Upper extremity	40 (23.9)
	Lower extremity	134 (80.2)
	Pelvis	20 (12.0)
ICP monitor, *n* (%)		137 (16.5)

*n* = Number; CPR = Cardiopulmonary resuscitation; ICP = Intracranial pressure probe.

**Table 4:** Distribution of 30-day complications.

	ESI	Non-ESI	*p*-Value
	711	40	
Infection, *n* (%)	294 (41.5)	15 (38.5)	n.s.
Pneumonia, *n* (%)	195 (28.8)	12 (34.3)	n.s.
Sepsis, *n* (%)	170 (24.1)	9 (23.1)	n.s.
Bacteremia, *n* (%)	85 (12.8)	3 (8.6)	n.s.
In-hospital mortality, *n* (%)	248 (34.8)	11 (27.5)	n.s.

*n* = Number; n.s. = not significant; ESI = Emergency Surgical Intervention.

## Data Availability

All data of this submission are available from the Dryad Digital Repository.
